# *In Mesopore* Protein Digestion: A New Forthcoming Strategy in Proteomics

**DOI:** 10.3390/molecules16075938

**Published:** 2011-07-15

**Authors:** Rocco Savino, Francesca Casadonte, Rosa Terracciano

**Affiliations:** Laboratory of Mass Spectrometry and Proteomics, Department of Experimental and Clinical Medicine, “Magna Græcia” University of Catanzaro, Catanzaro 88100, Italy

**Keywords:** mesoporous materials, protein digestion, catalysis, mass spectrometry, proteomics, nanotechnology

## Abstract

The conventional protocols for *in solution* or *in gel* protein digestion require many steps and long reaction times. The use of trypsin immobilized onto solid supports has recently captured the attention of many research groups, because these systems can speed-up protein digestion significantly. The utilization of new materials such as mesoporous silica as supports, in which enzyme and substrate are dramatically concentrated and confined in the nanospace, offers new opportunities to reduce the complexity of proteomics workflows. An overview of the procedures for *in situ* proteolysis of single proteins or complex protein mixtures is reported, with a special focus on porous materials used as catalysts. The challenging efforts for designing such systems aimed at mimicking the biochemistry of living cells are reviewed. Potentials, limitations and challenges of this branch of enzyme catalysis, which we indicate as *in mesopore* digestion, are discussed, in relation to its suitability for high-speed and high-throughput proteomics.

## 1. Introduction

Proteomic approaches have been increasingly applied to the study of protein networks and expression patterns in cells, tissues, and organisms, exploring their function at the molecular level. Over the last few years, proteomic research has stimulated the application of new techniques and experimental strategies, expanding its interface role to broad and diverse research areas of science and technology [1,2,3,4]. Mass spectrometry (MS) is considered the gold standard for the wide ranging applications of proteomics, becoming the method of choice for protein analysis and identification [5]. Among different possible strategies, two main prototypic mass-based workflows are mostly pursued in proteomics: *Differential Proteomics* and *Shotgun Proteomics.* In the case of *Differential Proteomics* the workflow involves the separation of proteins by the traditional 1- or 2-D gel electrophoresis (GE), followed by proteolytic digestion of protein band/spots of interest; the resulting digested mixture is then analyzed by MS for protein identification. *Shotgun Proteomics* is based on enzymatic digestion of complex protein mixtures, followed by extensive fractionation of peptides by multidimensional liquid chromatography (LC) and automated MS/MS analysis [6,7]. In this two proteomic workflows, proteolysis is performed according to two different protocols: *in gel* digestion [8,9] in the former, and *in solution* digestion [10,11] in the latter. Although used by the great majority of proteomic investigators, both these conventional digestion procedures need many steps, complex sample handling and exceedingly long digestion times, which all limit high-throughput protein identification. Therefore, new flexible and efficient tools are an urgent priority, in order to satisfy the demand for high-speed analysis. In the last years, we have assisted to a huge increase of *non*-conventional digestion strategies designed to overcome the major drawbacks encountered in conventional protein digestion procedures.

Among promising non-conventional procedures, irradiation by microwaves [12,13] or ultrasound [14], applied both to *in gel* and *in solution* protein digestion, have shown a dramatically improved efficiency in speed and sensitivity of proteolysis. Recently, successful *in solution* digestions of both single proteins and complex protein mixtures were achieved in 60 s using pressure cycling technology in the range of 5 to 35 kpsi [15]. Among the non conventional methods, the use of high-concentration trypsin immobilized onto solid support has recently captured the attention of many research groups, because these systems can significantly speed-up digestion, due to the possibility to work with enzyme-to-substrate (E/S) ratios higher than in conventional digestion [16,17]. Enzymes for protein digestion can be immobilized in different ways onto different kinds of favorable support (polymers, silica, porous silicon, mesoporous silica, agarose, nanofibers, *etc.*) in various formats or devices (beads, cartridges, capillaries, columns).

The so called *in column* digestion constitutes a new trend in current proteomics [18]. Proteolytic enzymes, usually trypsin, are chemically immobilized and a protein solution is infused and digested through the column. Most notably, immobilization on columns is conceived inside microfluidic systems [19,20]. The development of microfluidic enzymatic reactors, coupled on-line with mass spectrometers for proteomics applications, has demonstrated to be advantageous [21,22], for the possibility of direct coupling with LC and MS [23], which eliminates possible sample loss or contamination observed in manual handling. Therefore, protein digestion based on enzyme immobilized into microfluidic devices, through the integration of analytical processes into MS platforms, not only provides the rapid identification of proteins, but has also the great potential to contribute to a substantial improvement in overall sensitivity.

Most notably, trypsin is immobilized through covalent bonds. However, in order to covalently immobilize a protease to a specific support, many reaction steps are generally required. More than chemical immobilization, physical adsorption into a favorable support could dramatically simplify the experimental procedure. Recently, in such systems indeed, the immobilization of protease is accomplished *via* physical adsorption [24,25,26,27,28,29,30,31]. Mesoporous silica (MPS) materials constitute optimal supports for physical entrapment of enzymes. The use of proteolytic enzymes, in particular trypsin, immobilized on mesoporous materials has increasingly captured the attention of several research groups, because it could turn highly favorable for high-throughput proteomics. We report herein an overview of the procedures for *in situ* proteolysis of single proteins or complex protein mixtures, with a special focus on MPS materials used as “nanobiocatalysts”. The challenging efforts for designing such systems, aimed at mimicking the biochemistry of living cells, are reviewed. Potentials, limitations and challenges of this branch of enzymatic catalysis, which we indicate as *in mesopore* digestion, are discussed, in relation to its suitability for high-speed and high-throughput proteomic analysis.

## 2. Ordered Mesoporous Silica

**Scheme 1:**
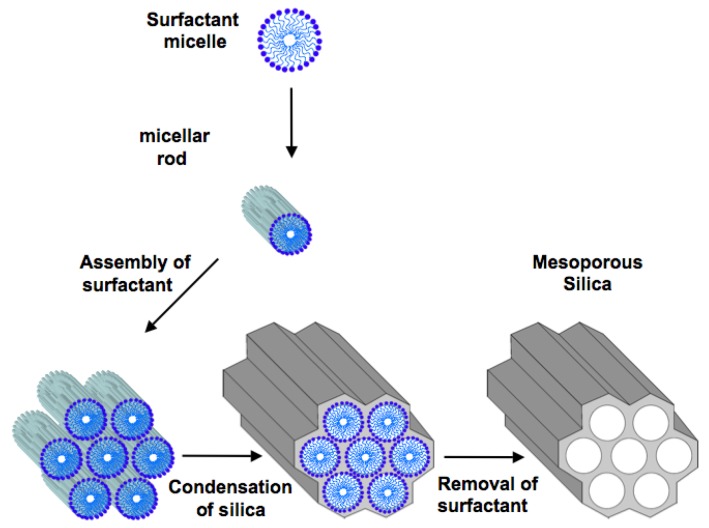
The synthetic route followed to obtain mesoporous silica materials. Surfactant molecules organize themselves, with their long chains, into micellar liquid crystals which serve as templates for the formation of mesopores. Then, when the silicate is added, it deposits itself around the micellar phase. A “cooperative self-assembly” takes place *in situ* between the templates and the mineral network precursors, leading to organized architectures: subsequent aggregate removal, for instance by calcinations or extraction, generates well-ordered mesopores, separated by amorphous silica walls.

According to IUPAC nomenclature, porous materials are divided into three classes: microporous materials with a pore diameter below 2 nm, mesoporous materials with a pore diameter between 2 and 50 nm, and macroporous materials with a pore diameter above 50 nm. Ordered MPS should not be confused with porous silica gel, which is commonly used as a stationary phase for chromatographic separations. The surface areas of commercially available chromatographic grade silicas are generally less than 500 m^2^/g, which is high relative to most materials [32]. Moreover, the wide range of pore sizes in silica gels makes their application in shape selective catalysis unfeasible [33]. Quite different, for their structural properties, are ordered MPSs characterized by regular arrangement of mesopores with narrow pore size distribution, high specific surface areas (up to *ca.* 1500 m^2^/g) and pore volumes (up to *ca.* 1.5 cm^3^/g), which render them ideal candidates as hosts for biomolecules [34]. In fact the high surface area allows a great accessibility to molecules; moreover, the regularity of porous arrays makes an effective control of molecular adsorption based on size easily feasible.MPSs, discovered for the first time by Mobil oil researchers in 1992, were synthesized by the hydrolysis and condensation of inorganic precursors (the sol-gel process) in the presence of surfactant micelles (templates) [35,36]. The possibility of synthesizing mesostructured silica materials with regularly sized pores, templating them by the mean of supramolecolar aggregate of surfactants, represents a starting point for the design of functional nanostructured materials. A synthetic route which is generally used for the preparation of these materials is described below and shown in Scheme 1. Briefly, during the synthesis, the long chains of the surfactant molecules organize into micellar liquid crystals, which serve as templates for the formation of mesopores. When the silicate is added, it deposits itself around the micellar phase. In many cases a “cooperative self-assembly” can take place *in situ* between the templates and the mineral network precursors, leading to organized architectures. Then, the aggregates of surfactant are removed by calcinations/extraction, generating well-ordered mesopores, separated by amorphous silica walls. While tetraethylorthosilicate (TEOS) is commonly used as the source of silicate [37,38,39,40], a great variety of surfactants with different alkyl chain length have been employed in order to both achieve different geometric mesostructures and modulate pore size [41,42,43]. Beck *et al.* were able to tailor the pore size from 1.5 to 4.5 nm by varying the chain length of quaternary trimethylammonium cations between 8 and 18 carbon atoms. The addition of organic molecules such as 1,3,5-trimethyl-benzene [36] or alkanes [44] allowed the increase in pore size up to 10 nm.

The most exploited and investigated MPSs are the M41S family members [Mobil Composition of Matter (MCM)-41 MCM-48, MCM-50] and the Santa Barbara Amorphous (SBA)-n type materials (SBA-1, SBA-3, and SBA-15) [45]. SBA materials show generally larger pore sizes (5–30 nm) in comparison to MCM (1.5–10 nm). In particular SBA-15 is commonly used because its uniform morphology can be easily controlled during the synthesis and for its exceptional capability in biomolecules adsorption and desorption [26].

Besides the possibility of tailoring both the shape and the size of the pores, it is also possible to modify the surface properties by introducing chemical functionalities, in order to provide specific, desirable features. Derivatization can be performed by post-synthesis (grafting methods) or by direct synthesis (co-condensation) [46,47]. In the former procedure, silica surface is functionalized through silylation reactions which take place on isolated and geminal silanols, using trichloro- or trialkoxyorganosilane and silylamines as organic precursors. In the latter, a co-condensation of siloxane and organosiloxane precursors occurs in the presence of the corresponding structure-directing agent. Although the co-condensation method leads to a more homogeneous surface coverage of organic groups on silica walls, better defined and hydrolytically more stable products are obtained when the grafting method is used [48].

MPS materials, with ordered pore structure, high surface area and high concentration of hydroxyl groups on the surface, have captured considerable interest for a diverse range of applications, such as catalysis, filtration and separation, molecular collection and storage, nanofluidics, medical imaging, drug delivery and sensors. We refer the reader to in dept literature on these topics here and below [49,50,51]. Recently, our group has explored a new application of MPS materials for proteomic analysis [52]. We demonstrated that MPS materials are able to harvest peptides from plasma, serum and other bodily fluids by the means of nano-sized porous channels with high surface area, which exclude large size proteins by a cutoff mechanism. Extracted peptidome is then profiled and analyzed by Matrix Assisted Laser Desorption Ionization-Time of Flight (MALDI-TOF) MS. The possibility to reveal complex fingerprints by this material platform represents a direct avenue for biomarker discovery in readily accessible body fluids [53,54].

Another proteomic application of MPS materials was pioneered by researchers of Fudan University in 2005 [25]. They demonstrated that MPSs can be used as efficient nanoreactors for protein digestion. However, the concept and the application of mesoporous materials as “natural microreactors” to accommodate catalyst entities such as metals, metal oxide nanocomposite, semiconductors, carbon materials and so forth were already known since the late ’90s [55,56].

The discovery of mesoporous materials has opened new horizons and opportunities to heterogeneous catalysis. The high surface areas and the ordered periodicity of the pores, with controllable dimensions and morphology, render mesoporous silicate optimal supports for a great variety of catalysts, from small catalysts such as metals, metal complexes, metal oxides to large molecule catalysts such as enzymes. In conclusion, due their above described features, MPSs offer a broad range of applications in many fields; in particular in the last years MPSs are increasingly exploited for proteomic analysis both in peptidome profiling both in assisted protein digestion. In the next section we will mainly highlight the use of ordered MPSs as supports for enzyme catalysts.

## 3. Mesoporous Silica for Supporting Enzyme Catalysts

Enzymes are highly selective biological catalysts, which are essential in living systems for their exquisite regio-, stereo- and substrate-specificity. Therefore, enzymes have found wide application in medicinal chemistry, organic synthesis and biotechnology, *etc*. However, the use of enzymes is subject to a number of limitations and drawbacks. For example, with few exceptions, enzymes are relatively expensive and, therefore, disposing of them after a single cycle of production is not economical. Moreover, enzymes have evolved to perform catalysis in very mild conditions, unless they are purified from rare extremophile organisms (which makes their production even more expensive). An obvious consequence is the general instability of the majority of enzymes towards heat, organic solvents, acids or bases, *etc.*, which limits their use in industrial applications, in which such harsh conditions are often the norm [57]. One possible solution to these issues is to immobilize the enzyme on a appropriate support. Not only does this facilitate the recovery and reuse of the enzyme, but in many cases, immobilized enzymes show a higher stability than free species. Therefore, a number of methods for the immobilization of enzymes, such as attachment to prefabricated carriers (by covalent or ionic binding, adsorption, etc.) or cross-linkage and entrapment (encapsulation), have been developed [58].

The performances of MPS substrates as inorganic support for proteins and, in particular, enzymes have been extensively studied. This field of research was opened by a report dating back in 1996, in which Diaz and Balkus studied the properties of a mesoporous material, MCM-41, as support for the adsorption of the four enzymes cytocrome c (Cyt c), trypsin, papain and peroxidase [59]. This pioneering work explored many of the main issues of the field. In particular, the influence of the protein size, relative to the material pore diameters, on the support adsorption capacity was first demonstrated. The same authors also explored the issue of enzyme leakage by reducing the pore diameter through silanation after enzyme adsorption. Finally, the preservation of catalytic activity of the adsorbed trypsin was also demonstrated [59]. Since then, over 150 articles have appeared in the literature, which have explored all aspects of protein-mesoporous material interaction, including relative size of the mesopores and the protein, mesoporous material particle size and morphology, influence of the isoelectric points of mesoporous silicates and protein on the adsorption process, functionalization of the silicate surface, optimization of the conditions for immobilization and the nature of the interactions between the proteins and the support. Of fundamental interest are the issues of preservation of enzymatic activity after immobilization and, in particular for industrial application, of maintenance of enzymatic activity under harsh conditions (heat, organic solvents, acids or bases) and of reusability and enhanced stability of the immobilized proteins. All these issues have been summarized in excellent reviews [58,60] and will not be repeated here. In summary, over 60 different proteins were immobilized on mesoporours materials of various kind, over 50 of these proteins were enzymes and the catalytic activity of the immobilized enzyme was demonstrated with few exceptions [60,61,62,63,64,65,66,67,68,69,70,71,72,73,74,75,76,77,78,79]. Studies comparing the catalytic activity of the free enzyme to that of the immobilized form were published for over 30 different enzymes. Interestingly, the catalytic activity of the immobilized form was determined to be higher than that of the free enzyme in 20 cases. The immobilized form was reported to be less active than the free enzyme in 13 cases; however, in this smaller group, the decreased catalytic activity was compensated by an enhanced stability in 10 cases, seven of which with the additional advantage of the demonstration of the re-usability [60,61,62,63,64,65,66,67,68,69,70,71,72,73,74,75,76,77,78,79]. It can therefore be concluded that not only the use of mesoporous materials as enzyme support is becoming more and more popular in the scientific community, but these materials are beginning to demonstrate very interesting potential applications in biocatalysis.

## 4. Mesoporous Silica as Supports for Protein Digestion in Proteomics

### 4.1. General Overview of the Field

In principle, an enzyme accelerates a specific biochemical reaction by establishing interactions with the substrates inside its structurally pre-organized active-site. In such an environment, binding interactions with the transition state are favored over the ground state [80].

Among other functions, trypsin is a digestive enzyme which cleaves polypeptide chains at positively charged arginine/lysine residues [81]. In proteomics, this specificity offers several advantages. In fact, lysine and arginine are presents in proteins primary sequences approximately every 10–12 aminoacids; consequently, tryptic fragments with an average size of 800–2,000 Da are generated, which can be measured with modern mass spectrometers with high accuracy. In addition, in tandem mass experiments, a favored homogeneous fragmentation of tryptic fragments occurs by collision activated dissociation [18]. Trypsin belongs to the family of serine proteases, which covers one third of all known proteolytic enzymes and it is so named due to the presence of a serine residue (Ser) in the enzyme active site [82]. The catalytic triad of Asp 102, His 57, and Ser 195 residues is responsible of the hydrolysis of the peptide bond. In particular, the nucleophilic hydroxyl O atom of Ser 195, utilizing His 57 as a general base (stabilized by Asp 102), attacks the carbonyl of the amide bond in the substrate, to give an oxyanion tetrahedral intermediate, stabilized by a positively charged pocket (oxyanion hole) within the active site. After the collapse of the tetrahedral intermediate, an acyl-enzyme intermediate and a N-terminus free polypeptide fragment are generated. The attack of a water molecule to the acylenzyme intermediate forms a second tetrahedral intermediate, which collapses generating new C-terminus in the substrate [83]. Trypsin, with its highly efficient fold that couples catalysis and regulatory interactions, is an extraordinary and great machine, considering the nanoscopic scale on which it can distinguish structural substrate rearrangement. As others enzymes, trypsin exhibits high catalytic activity and selectivity under mild conditions, but it is difficult to handle and it is susceptible of inactivation by pH changes, organic solvents and high temperature. Indeed high temperature and long incubation time may lead to deamidation, transpeptidation, metionine and cysteine oxidation and non specific cleavage. Furthermore, autolysis may also be observed [18]. Therefore, in order to avoid the appearance of interfering autolysis peptides, low trypsin-to-substrate ratio has to be kept. As consequence, the kinetic is slow and digestion requires several hours to proceed. Additionally, in current proteomics workflows, where complex biological protein mixtures are completely digested, another drawback may be encountered due to low-micromolar substrate concentrations. As just anticipated above, a porous support can offer both a robust physical-chemical environment for trypsin and thermal stability, enabling it to be used in cyclic operations.

The escalation of proteomics, concomitantly with the urgent demand in term of bio-analytical and bio-chemical tools, has merged different and separated research areas into a multidisciplinary approach. Remarkable examples of this trend are given by the great contributions brought to proteomics from organic, analytical and material chemistry. Material sciences in particular offer to proteomics not only a vast arrays of chromatographic phases for fractionation and separation purposes, but also nanotextured surfaces for the enhancement of proteomic analysis via MS [54,84]. Over the last decade, MPSs have been increasingly studied as support for enzyme immobilization for their potential applications in biocatalysis [60]. Specifically, the interest to catalyze proteolysis by MPS supports is matured only in the last six years, maybe due to the urgent demand of new strategies for high-throughput proteomic analysis. Trypsin, confined in the inorganic nanospace of MPS, provides increased cleavage efficiency. The increased bioactivity of trypsin confined into a mesoporous channel over the free enzyme may be due a remarkable local enzyme–substrate enrichment within the nanopores as reported in different studies in this field [25,26,27,29,30,31].

### 4.2. Substrate Pre-Adsorption

**Scheme 2:**
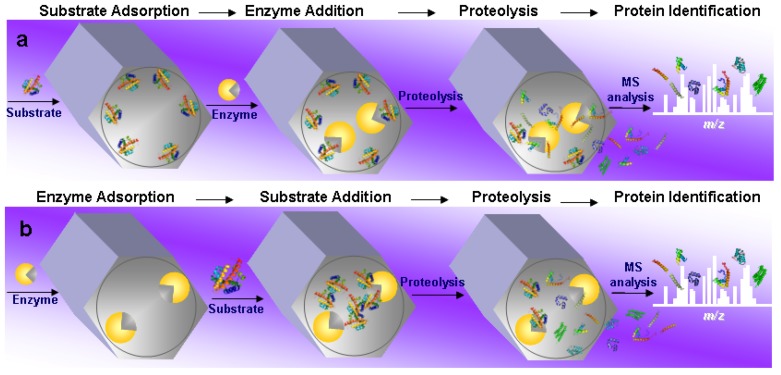
Two common workflows for *in mesopore* protein digestion procedure. (**a**). The substrate is adsorbed from solution into a solid mesoporous support. The enzyme is then added and adsorbed in the mesopores. The substate is digested inside the mesopore, then peptidic fragments are released from the support to the bulk solution and analyzed by MS; (**b**). The enzyme is adsorbed from solution into a solid mesoporous support. The substrate is then added and adsorbed into the mesopores. Digestion occurs inside the mesopore, then peptidic fragments are released from the support to the bulk solution and analyzed by MS.

The first example of “*in mesopore*” protein digestion using MPS materials was reported by Fan *et al.* [25]. The authors demonstrated the highly efficient proteolysis carried out on the enzyme-substrate model trypsin-myoglobin, using SBA-15 as a nanoreactor. Myoglobin (see Table 1 for MW, pI and protein dimension) was firstly adsorbed into SBA-15 mesopores, then proteolysis occurred after the addition of trypsin (Scheme 2a). In particular, in a first step, the adsorption of myoglobin was accomplished in acidic conditions. Then the beads were centrifuged and washed before the addition of the enzyme in an E/S = 1:2. In the last step, the beads were centrifuged and washed, then incubated in aqueous bicarbonate solution (pH = 8.2). Therefore, the proteolysis of the entrapped myoglobin was activated by changing the pH of the buffer from acidic to basic conditions. In this last step, the digestion products were released from the SBA-15 meso-reactor and subsequently analyzed by MALDI-TOF MS. In order to monitor the efficiency of the digestion, protein identification was performed by MALDI-TOF MS, based on peptide mass fingerprinting (PMF). As control, an experiment was also carried out without MPSs, with free trypsin in solution, using the same experimental conditions of the *in mesopore* digestion. Interestingly, in this first study, the authors investigated how different factors (substrate-enrichment, surface-character and macrostructure) could affect the efficiency of the *in mesopore* digestion. MPS SBA-15 materials, with one-dimensional mesochannels packed in a two-dimensional hexagonal structure, of different particle size and surface character, were examined. Among the different samples investigated, SBA-15-SH, the thiol-functionalized material, with a pore diameter of 8 nm, demonstrated the highest catalytic efficiency: Eight tryptic peptides, generated from the *in mesopore* myoglobin proteolysis, were analyzed by MALDI-TOF MS, resulting in a sequence coverage of 58% in about 10 min after trypsin addition to the beads. On the contrary, in the control experiment with free trypsin, only three peptide matches were obtained in 12 h with a sequence coverage of 27%. The increased efficiency of proteolysis inside the nanoporous silica beads over the one performed with conventional *in solution* procedure was ascribed to the highly efficient enrichment of the substrate and of the protease inside the porous support. Other detailed conditions for this model system are given in Table 2.

**Table 1:** Molecular weight (MW), isoelectric point (pI)and size of model proteins used for “in mesopore” digestion.

Protein	MW (Da)	pI	Dimension (nm)
Myoglobin	17000	7.0	2.1 × 3.5 × 4.4 ^a^
Cytochrome *c*	12384	10.0	2.6 × 3.2 × 3.3 ^a^
Bovine Serum Albumin	66400	4.7	5.0 × 7.0 × 7.0 ^a^
Ovalbumin	42700	4.9	7.0 × 4.0 × 5.0 ^b^
Conalbumin	76000	6.0	5.0 × 5.6 × 9.5 ^a^
Lysozyme	14388	10.8	1.9 × 2.5 × 4.3 ^a^
Trypsin	23400	10.5	*ca.* 3.8 ^b^

^a^ protein dimensions from reference [34]; ^b^ protein dimensions from reference [60].

Later, the some group, optimized the conditions of their previous protocol, obtaining 19 tryptic peptides with 98% of sequence coverage of myoglobin in 15 min after the addition of trypsin [26]. They varied the previous procedure by increasing the incubation time for substrate adsorption inside MPS, by eliminating the washing steps after the substrate adsorption into MPS and by increasing the digestion time. The elimination of washing step was adopted in order to reduce substrate loss. Additionally, they used SBA-15 material with a pore diameter of 7 nm. The MALDI-TOF MS analysis revealed 19 myoglobin tryptic fragments (corresponding to a sequence coverage of 98%) 15 min after the addition of trypsin. On the contrary, *in solution* digestion performed in the same time span, originated only few myoglobin fragments, unsatisfactory for unambiguous protein MS identification. The sequence coverage of myoglobin increased to 67% only after an overnight digestion. As the authors pointed out, with their procedure it was possible to effectively concentrate a diluted protein/substrate solution through the confinement into the nanospace of MPSs, prior to digestion. The increased protein concentration prior to digestion improved the enzyme kinetics, while, in absence of MPS, the *in solution* digestion took place in more diluted conditions for both the substrate and the enzyme.

**Table 2:** A comparison of mesoporous materials performances for *in mesopore* protein digestion.

MPS Material (mg)	Pore size (nm)	Substrate (amount digested)	E/S (w/w)	Digestion time	N of prot.frag (S/N)	Seq. Cov.	Ref.
SBA-15-SH (2.5)	8	Myoglobin (50 μg)	1:2	10 min	8 (70)	58%	[25]
SBA-15	7	Myoglobin (179 ng)	1:2(2:1)	15 min	19 (20)	98%	[26]
(2.5)		Cyt c (124 ng)	1:2(2:1)	15 min	14 (20)	84%	
FDU-12	17 ^a^	Myoglobin (50 μg)	1:2	15 min	12 (>80)	84%	[27]
SBA-15	8	Myoglobin (50 μg)	1:2	15 min	NR(>80)	31%	
MCF	15 ^b^	Myoglobin (50 μg)	1:2	15 min	NR(>80)	22%	
(2.5)		Ovalbumin (45 μg) /Conalbumin (15 μg) /Cyt c (1 μg)	1:2	15 min		42%	
		Nuclear proteins mouse liver cell (10 μg)				26%	
						58%	
			1:8	30 min			
HMS(CNS)	18	Cyt c (600 μg)	1:3	20 min	11 (NR)	63%	[29]
(1)		Myoglobin (600 μg)	1:3	20 min	12 (NR)	NR	
		Cytoplasm of human liver tissue (5 μg)	1:3	20 min			
SBA-15 SH	5.7	Cyt c/BSA ^c^	1:15	10 min ^c^	8 (NR)	46%	[30]
(3)		Lysozyme/BSA ^d^	1:15				
		Myoglobin/BSA ^d^	1:15				
		Human serum (150 μg)	1:15	60 min			
SBA-15 (1)	4.3	Myoglobin (20)	1:3	1 min	7 (>50)	43%	[31]
SBA-15 (1)	6.3	Myoglobin (18)	1:3	1 min	2 (>50)	17%	[31]
SBA-15 APTES (1)	4.3	Myoglobin (16.9)	1:3	1 min	7 (>50)	60%	[31]
SBA-15 AAPTES (1)	4.4	Myoglobin (18)	1:3	1 min	21 (>50)	100%	[31]

^a^ three-dimensional interconnected nanopore networks in which ultra-large nanopores (27 nm) are connected by expanded entrances (17 nm); ^b^ disordered mesocellar foams with large cavities (27 nm) and wide entrance (15 nm); ^c^ the number of proteolytic fragments and sequence coverage is referred to Cyt c (in a binary system of Cyt c and BSA); ^d^ the number of proteolytic fragments and sequence coverage is reported at 10 min; only for the pair Cyt c and BSA, anywhere for 1 hour of digestion the number of proteolytic peptides and the coverage is reported for all three pairs of proteins [30]; NR: not reported; Cyt c: Cytochrome c.

With the same procedure described in the above mentioned studies and illustrated in Scheme 2 panel a, the same research group from Fudan University investigated the efficiency of a newly synthesized mesoporous silica material FDU-12 [85,86] as meso-reactor for proteolysis [27]. The main feature of FDU-12 was the highly ordered unique three-dimensional nanostructure in which ultra-large nanopores (27 nm) are connected by expanded entrances (17 nm). The authors demonstrated the efficiency of FDU-12 nanoreactor over *in solution* digestion, not only for individual proteins, but also for protein mixtures. The conditions and results are reported in Table 2. Interestingly, in this study the Authors reported the first example of *in mesopore* digestion of a real biological sample. In fact, a nuclear protein fraction isolated from mouse liver cells was digested inside the FDU-12 reactor. As control experiment, an *in-solution* digestion was performed on the native protein extract in the same conditions used for the nanopore reactor, *i.e.*, without pre-denaturation. From micro-LC MALDI-TOF/TOF analysis more peptide signals from *in-mesopore* digestion were detected in comparison to that from *in-solution* digestion. In fact, 98 proteins were confidently identified from the *in-mesopore* tryptic profile compared to only six proteins unambiguously identified from the *in solution* digestion. This superior efficiency of proteolysis within MPS nanopores without pre-denaturation over the *in solution* proteolysis (whose procedure normally includes a denaturation step) implies that, when the substrate is adsorbed inside the mesopores, it may undergo to an unfolding process making its cleavage sites fully accessible to the enzyme. The putative interaction between basic aminoacid residues of the protein substrate with the silanol groups present on the MPS walls may trigger the unfolding process. Remarkably, it is worth mentioning, in this study, the exploration of some mechanistic aspects of *in mesopore* digestion, in particular the postulated substrate unfolding within mesopores was supported by experiments based on Raman spectroscopy. Interestingly, myoglobin confined in the mesospace of the FDU-12 material showed a Raman spectrum very similar to that of myoglobin after thermal denaturation. From the analysis of these experimental evidences, the authors hypothesized in the “reagent-free structural unfolding” the basis for an effective entrapment, assumed as the key element of an efficient proteolysis. Additionally, a structural factor was also found to effectively contribute to proteolysis. In fact, the authors compared the results of *in mesopore* proteolysis (in term of sequence coverage) for three different mesostructured materials: FDU-12, SBA-15 [87], and MCFs [88]. As illustrated in Table 2, for the same amount of myoglobin substrate with the same E/S and in the same experimental conditions, the best results were obtained for FDU-12, while only 31% and 22% of myoglobin sequence was covered from the proteolytic peptides generated from SBA-15 and MCFs assisted proteolysis, respectively. Therefore, the authors concluded that the enhancement of proteolytic activity in FDU-12 over SBA-15 and MCFs was due to their structural differences. In particular, the well organized cavities of FDU-12 facilitated the mass diffusion and transportation through the 3D interconnected mesopores. This process was instead less favored in the 2D hexagonal packing 8 nm channels of SBA-15 or even hindered in the disordered mesocellar structure of MCFs.

In conclusion, in this subsection different examples of *in mesopore* digestion have been reported in which substrate adsorption is performed before enzyme addition. SBA-15 and FDU-12 materials (with larger pore size) used in the above described experiments revealed their excellent ability in enriching protein substrates, thus facilitating their digestion inside their inner surface after enzyme diffusion. In particular SBA-15, opportunely functionalized with thiol groups, improved its adsorption efficiency towards substrate, while the larger pores in FDU-12 material, combined with its unique three dimensional mesostructure, were demonstrated to be useful for adsorption and consequent digestion of complex protein mixtures.

### 4.3. Enzyme Pre-Adsorption

The above described mesoporous reactors for protein digestion are based on the procedure illustrated in Scheme 2 panel (a) *i.e.*, substrate adsorption into mesopores, followed by enzyme addition. On the contrary, we will describe in the next pages a similar procedure with a slightly modified workflow based on enzyme immobilization by physical adsorption inside the MPS materials before substrate addition (Scheme 2 panel b).

Quiao and colleagues proposed a nanoporous reactor for efficient proteolysis based on cyano-functionalized mesoporous silicate (CNS) with a pore diameter of 18 nm [29]. Their preliminary data revealed a high adsorption capacity of CNS for trypsin (18 micromoles per g) [89]. They estimated CNS as a good support for trypsin immobilization, substrate capture and *in situ* proteolysis. Two models proteins, Cyt c and myoglobin (Table 1), but also a complex biological sample, extracted from the cytoplasm of human liver cells, were enzymatically digested inside the CNS-nanoreactor. In order to assess the enzyme loading necessary to guarantee the optimal substrate digestion and identification, different amounts of trypsin were immobilized on the mesoporous support. The immobilization of enzyme on CNS was performed by mixing the trypsin solution with mesoporous beads suspension in phosphate buffer at pH = 6.5, for 16 h. At pH 6.5, trypsin (pI = 10.5, Table 1) is positively charged, therefore a favorable interaction with the negatively charged CNS walls of mesopores was hypothesized by the authors. The CNS-trypsin particles were then separated by centrifugation, washed with ammonium bicarbonate (pH 7.8) and then immersed for 20 min in the substrate solution (different concentration from 2 up to 200 ng mL^−1^ in ammonium bicarbonate at pH = 7.8), assuring an E/S of 1:3. When the extract from human liver cells cytoplasm was digested, the protein concentration in the solution was 1 μg μL^−1^. For control experiments, the conventional *in solution* digestion was performed with E/S of 1:30, denaturing the substrate at 100 °C for 5 min in ammonium bicarbonate buffer, before adding the protease, then, the solution of enzyme and substrate was incubated for 12 h at 37 °C. As reported in Table 2, the best performances for CNS-mesoreactor were obtained when 1 mg of CNS beads were loaded with 200 μg of trypsin and incubated in 600 μg of Cyt c; digestion led to the identification of Cyt c by PMF (11 peptides matches by MALDI-TOF MS) with 63% of sequence coverage. Instead, a sequence coverage of 59% was observed for Cyt c after overnight *in solution* digestion. Moreover, in this study the authors found that proteolysis was still efficient also when the concentration of proteins was reduced to 2 ng μL^−1^, with six peptides of myoglobin and six peptides of Cyt c identified after a digestion time of 20 min.

Very recently, Min and colleagues [30] described a size-selective proteolysis on the mesoporous thiol-modified SBA-15 material. In order to demonstrate the “size-selective” proteolysis, size-dependent adsorption performance of mesoporous SBA-15-SH was proved. Four model proteins Cyt c, lysozyme, myoglobin and BSA, with different sizes, were chosen. In Table 1 molecular weights, isoelectric points and sizes of model proteins used for *in mesopore* digestion experiments are reported. Each protein was incubated with SBA-15-SH. UV absorbance experiments were performed to determine the amounts of protein adsorbed as function of time. Cyt c, myoglobin and lysozyme, with sizes smaller than the pore diameter of SBA-15-SH, were rapidly adsorbed (10 min for Cyt c and lysozyme and 5 h for the bigger myoglobin) while only a little adsorption was observed for BSA in 24 h of incubation. Having demonstrated substrate size-dependent adsorption, the authors focused on optimizing the conditions for enzyme immobilization. In particular, trypsin was physically adsorbed into SBA-15 MPS, modified with thiol, with pore size of 5.7 nm. The authors demonstrated that the presence of thiol groups on SBA-15 silica walls was able to minimize the leaching of immobilized trypsin, ascribing that to the favorable interaction between the -S-S- exposed on the surface of trypsin and thiol groups on MPS. In a second set of experiments, the size-selective proteolysis of SBA-15-SH with immobilized trypsin was demonstrated by digesting protein binary mixtures composed of BSA and either Cyt c, lysozyme or myoglobin with concentration of 5 nmol mL^−1^ for each protein and with an E/S ratio of 1:15 (see also Table 2 for additional details). As control experiment, the protein pairs were also digested by free trypsin in solution. The analysis of MALDI-TOF mass spectra revealed that, when the digestions were performed inside the mesoreactor, tryptic peptides derived from small size proteins were always prevalent over the tryptic fragments derived from BSA. On the contrary, peptides derived from BSA dominated the MALDI-TOF mass spectrum when free trypsin was used in control experiments. Therefore, the authors concluded that digestion of low-MW proteins by trypsin immobilized on SBA-15-SH was “size-dependent”, as a consequence of the size-exclusive interaction of the mesopores against the higher MW BSA protein. Additionally, the sequence coverage of each protein was also analyzed by nano-LC-MS/MS. Interestingly, the authors noticed that, for prolonged incubation times, the size-exclusive effect of the enzyme reactor toward high-MW proteins weakened. A likely explanation of this phenomenon resides in the potential adsorption of trypsin onto the external surface of SBA-15-SH, which therefore can digest high-MW proteins which are not totally excluded from the adsorptive process. Consequently, shortening the digestion time constitutes a possible way to avoid the extra digestion of high-MW proteins. Regarding to the efficiency of proteolysis for the protein pair BSA/Cyt c, a sequence coverage of 5.27% and 45.71%, respectively, was obtained in 10 min of incubation. These values increased to 16.64% and 74.29% after 60 min of incubation. This reactor was also used for digestion of a complex biological sample, such as human serum. In order to demonstrate the selectivity of proteolysis of the enzymatic mesoreactor toward the low molecular weight proteins of serum, the tryptic peptide mixture was analyzed by nano-LC-MS/MS, after 1 h of digestion. The data, in term of spectral count percentage versus MW of identified proteins, were compared to those obtained using free trypsin in identical conditions. As expected, the proteins with MW up to 40 kDa dominated the spectral counting distribution in the case of trypsin/SBA-15-SH reactor; on the contrary MW superior to 60 kDa and mainly located in the range 60-80 kDa were prevailing in the spectral counting distribution when free trypsin was used. Although the size-dependent adsorption performance of mesoporous material was previously demonstrated by others for model proteins [90] and also by our group both with models proteins [91] and with human plasma [52] and urinary proteins [53], the originality of this study mainly resides in the “size-selective proteolysis”. This represents a quite novel application which could be quite promising in proteomics, designed for retrieving the peptide information from low-MW proteins selectively enhanced by the size-selective proteolysis.

As a part of an ongoing project aimed at the development of convenient and sensitive procedures for proteomics applications [52,53,54], our group has recently rationally designed and developed a new enzymatic mesoreactor for ultrafast protein digestion [31] using the workflow shown in Scheme 2, panel b. In particular, proteolytic fragments of myoglobin were obtained as quickly as 1 minute from the addition of the protein to the trypsin adsorbed on *N*-(2-aminoethyl)-3-aminopropyl derivatized SBA-15 MPS, with pore size of 4.4 nm, giving a 100% sequence coverage. In this study, we investigated the effects of the pore size and of the surface modification of SBA-15 MPS on the efficiency of both adsorption and proteolysis. To this end, MPS SBA-15 together with *N*-(2-aminoethyl)-3-aminopropyl and aminopropyl (indicated as AAPTES and APTES, respectively) functionalized derivatives were prepared with pore size of about 4 nm. Another support, SBA-15, with larger pore size (6 nm), was also synthesized to monitor the effects of pore enlargement (Table 2). The design of such mesoreactor was based on the considerations described below. In order to obtain a simple and fast procedure for fabrication of an efficient enzyme reactor, we excluded the use of covalent bonding between the enzyme and the support, to avoid long complicated synthesis steps. Moreover, the chemical immobilization of trypsin into a mesoporous support, while reducing enzyme leaching from the support itself, has been reported to modify the conformation of the enzyme with reduction of catalytic activity [34,58]. Recent studies suggested that a stable enzyme adsorption is achieved when mesostructured material with a pore size close to the enzyme dimension are employed [60,92]. Therefore, to obtain a well-fitting physical entrapment, we synthesized SBA-15 mesoporous supports with pore dimensions of about 4 nm (Table 2), just slightly larger than the diameter of trypsin (Table 1). This ensured not only highly effective trypsin adsorption, with reduction of enzyme leaching, (which occurs when pore dimensions are much larger than enzyme), but also shortened adsorption times from several hours to few minutes. In ten minutes, a proteomic amount of roughly 0.3 nmol of trypsin was adsorbed per mg of MPS for both SBA-15 APTES and SBA-15 AAPTES; the same amount was loaded onto SBA-15 (4 nm) in 2 min and onto SBA-15 (6 nm) in only 1 min. It is worth mentioning that, in comparison to the other published immobilization procedures, our protocol allows a quite significant reduction of time, because we stopped the enzyme adsorption process before the equilibrium was reached. For example the cyano-functionalized mesoreactor (with a pore diameter of 18 nm) reported by Quiao and co-workers required a loading time of 16 h at room temperature [29], while 6 h of incubation were required for immobilizing trypsin on SBA-15-SH (pore size 5.7 nm) as reported by Min and colleagues [30]. Our controllable “trypsin-low-charging procedure” was finely tuned for proteomic analysis, where concentrations down to femtomoles of proteins have to be analyzed. We observed the lowest proteolytic activity for the un-functionalized SBA-15 (6 nm), in comparison to other SBA-15 (4 nm) materials (Table 2). In accordance with previous studies [25,29,93], a possible explanation of this phenomenon is that, after enzyme immobilization, myoglobin diffusion and accommodation inside the pore occurs in a more limited space, resulting in a partial substrate unfolding, with increased exposition of accessible cleavage sites. This partial substrate unfolding might occurs at lower efficiency when the pore diameter is larger.

In order to investigate the effects of the pore size and of the surface modification of SBA-15 MPS on the efficiency of proteolysis, we compared the digestion products released from SBA-15 without functional groups with those released from SBA-15-AAPTES and SBA-15-APTES, with the same pore diameter. In the first minute, tryptic peptides released from mesoreactors lead to a sequence coverage for myoglobin of 43%, 60% and 100% for un-functionalized SBA-15, SBA-15-APTES and SBA-15-AAPTES, respectively. This trend was also confirmed for the following reaction time courses, so at three minutes we observed a sequence coverage for myoglobin of 74%, 81% and 100%, for SBA-15, SBA-15-APTES andSBA-15-AAPTES; respectively; at five minutes the sequence coverage was of 83%, 88% and 100%, in the same order. Therefore, we concluded that the presence of the amine groups could be beneficial for the catalytic reaction. Interestingly, the length of the functional group (APTES: 0.5 nm, AAPTES: 0.8 nm) correlates with the increased performance. It is well established that the electrostatic stabilization of the charge in the oxyanion hole plays a crucial role in the mechanism of serine proteases activity [83]. Interestingly, the oxyanion hole is located on the external enzyme surface [94] and it is therefore easily accessible. Hence, it is temping to speculate that a potential mechanism might involve a role of basic groups, in particular the longer AAPTES, in an electrostatic stabilization of the charge in the oxyanion hole. It is worth noticing that recent studies envisage cooperative interaction of such functional groups among organo-functionalized MPS that give rise to enhanced catalytic activity [95].

As illustrated in all the studies reported in this section, excellent protein digestion yields were achieved with *in mesopore* digestion over *in solution* digestion. We therefore conclude that the elimination of several steps, generally considered essentials in the case of *in solution* digestion (for instance substrate denaturation, alkylation, overnight incubation), and the rapidity of proteolysis render the *in mesopore* digestion a suitable tool to simplify proteomic workflows presently in use. In addition, mechanistic factors at the basis of *in mesopore* digestion have also been investigated: It is clear that the field is at its infancy, the few published reports illustrated here are exploratory and further in depth studies are required to better understand and discover the great potential of MPS in proteomic analysis.

### 4.4. Factors Affecting in Mesopore Proteolytic Efficiency

While the effect of a functional group is specific for each chemical functionality grafted on the support, a general issue is the relationship between pore size and enzyme dimension/activity. The published data do not allow one to establish if *in macropore* digestion is more efficient than *in mesopore *digestion or *vice-versa*. Although it is difficult to compare the performances of different silica nanoreactors since diverse structures, geometries and experimental conditions are used, undoubtedly, the data shown by Quian and colleagues [96] for a macroporous support such MOSF (macroporous ordered siliceous foam) with pore size of 100 nm, also demonstrated an improvement compared to *in solution* digestion. In fact, when macroporous silica materials were used as catalysts, a better performance was observed, both for model proteins and for a complex protein mixture, in comparison to the conventional *in solution* digestion. The same group has later developed a polyfunctional device/macroreactor both for digestion and selective adsorption of glycopeptides. The same MOSF material, with same pore size (100 nm), functionalized with boronic acid, was used to fabricate a device. In this boronic derivatized macroporous silica material, after digestion, glycopeptides were selectively captured based on the affinity between boronic acid and glycol groups, while the non-specific peptides were released to the solution, or further purified by un-functionalized MOSF and/or NH_2_-MOSF materials [97].

As a matter of fact, to date only a few studies have been performed to assess the factors which regulate chemistry in nano-confinement. A great input to the field reviewed here might be provided by an interesting kinetic model for the macropore-confined proteolysis, recently described by Bi and colleagues [98]. To describe *in macropore* proteolysis, the authors assumed a multistep process, constituted by ingress, subsequent diffusion of substrate inside the macropore, enzyme-substrate encounter and substrate cleavage, diffusion in the macropore of peptides generated from substrate cleavage, further cleavage and, finally, release of peptides from the macropore. On the other hand, for conventional *in solution* digestion, they invoked a sequential proteolytic mechanism, in which the enzyme hydrolyzes the amide bond of a protein or of a peptide, generating peptidic fragments which diffuse away from the enzyme, back to the bulk solution. Therefore, the probability that the proteolytic fragments will encounter the enzyme in the bulk solution should be much lower than that in the macropore. In this case, the entrapment effect plays a crucial role in the kinetics of the proteolysis. Myoglobin was used as model substrate together with angiotensin 1, and ACTH (1–14), which contain one or two active digestion sites, respectively. Both conventional *in solution* digestion and *in macropore* digestion were performed, maintaining the same E/S ratio to 1:30. MOSF with pore size of 100 nm, pre-loaded with trypsin, was used for in macropore digestion. The Authors estimated a substantial reduction in reaction time of roughly 40 times for myoglobin digested in macropore, on the basis of proteolytic fragments (24 and seven for *in macropore* and *in solution* digestion, respectively), analyzed by MALDI-TOF MS after 30 min of reaction. Elaborating the kinetic equations, they observed that in addition to the entrapment (or nanoconfinement) effect, the effectiveness factor η is another key factor that may influence the kinetics in macropores. The factor η is the equivalent of the effectiveness parameter used for porous media [99]; which depends from the interaction between the porous support and the substrate. In fact, the rate of the reaction depends also by the equilibrium between substrate diffusion and *in-pore* reaction. In particular, the absence of specificity between the support and the substrate (η < 1), the overall kinetics is limited by diffusion and the enzyme reaction cannot run at its maximum rate. On the contrary, when the affinity between porous support and substrates is strong (*i.e.*, electrostatic interactions, hydrophilic/hydrophobic interactions or chemical interactions), the effectiveness factor η is ≥1, the diffusion is very fast, therefore the overall kinetics is limited by the digestion rate. It could be interesting to assess if this model could be adapted and revisited for *in mesopore* confined proteolysis.

An important issue of this field is the proteolytic activity/stability of enzyme, in this case trypsin. The fundamental question which should be asked is: “how much physical adsorption of trypsin in a mesoporous support stabilizes its activity?” Studies on the activity and stability of MPS-immobilized trypsin have been done, in particular, storage stability, reusability or influence of impurities in trypsin preparations on enzyme uptake have been reported by Goradia and colleagues [89]. In this study, the activity of trypsin immobilized on different MPS, was found 10–20 times higher than that of the free enzyme; moreover, MPS-immobilized-trypsin was also found stable for 4–6 weeks when stored at 4 or 25 °C. Finally, it was demonstrated that this system could be reused for up to six cycles to catalyze the hydrolysis of *N*-α-benzoyl-dl-arginine-*p*-nitroanilide.

Recently, Manyar and Colleagues investigated on the catalytic activity of porcine pepsin physically immobilized into SBA-15, in which APTES was grafted for reducing pore openings thus minimizing pepsin leaching [100]. The authors observed an high catalytic activity for the immobilized pepsin similar to that of the free enzyme on the substrate *Z*-L-glutamyl-L-tyrosine dipeptide. On the contrary a low catalytic activity was detected when a larger substrate such hemoglobin was used. In fact, unlike *Z*-L-glutamyl-L-tyrosine, haemoglobin, with dimensions higher than the SBA-15 pores used in this study, cannot diffuse inside the SBA-15 mesopores. Finally, the authors found that the pepsin-SBA-15 APTES bioreactor was recyclable up to four cycles without any loss of activity and could be effectively reused. Besides trypsin and pepsin, other proteases like papain or chymotrypsin are routinely used in proteomic protocols. Interestingly, there are reports describing the immobilization of papain or chymotrypsin in mesoporous supports [101,102] in which the enzymatic activity was monitored using small molecules or protein model substrates: It would be very attractive to start from the preliminary data reported in the above cited studies and test these bioreactors for digestion of samples to be studied in proteomic analysis. Enzymes are relatively expensive and, therefore, discarding them after a single use is not economical. Although in the examples reviewed the amount of enzyme is generally higher than in conventional digestion, the possibility to reuse the mesoreactor for several cycles might amortize the high costs of the enzyme. In order to be successfully reused, immobilized trypsin must have high activity, high stability, and high resistance to autolysis and also to proteolysis by other proteases often found in various kinds of proteomic samples. The high activity/stability of trypsin inside all the silica mesoreactors described in this review was proved by its high efficiency in proteolysis (Table 2). However, only in one of the studies summarized in Table 2, trypsin reusability for protein digestion was assessed [27]. In particular, the reusability of the protease was tested monitoring the activity after repetitive digestions by MS analysis of peptide recovery over 16 days: Only a little decrease in activity was observed.

A trend common to all the meso-reactors described here is the preferential choice to select physical adsorption of enzyme/substrate, which is simple and easy to perform in comparison to long and complex immobilization procedures. However a possible drawback encountered in this case might be the leaching of enzyme out from the mesopores, when a weak electrostatic interaction occurs between host and guest. In order to prevent leaching, covalent binding of the enzyme to the support has been explored, discovering however that these procedures might lead both to a loss in enzyme activity [24,34] and also to pore blocking effects by irreversible binding of the enzyme to pore entrance sites [103]. Although several immobilization procedure have been developed in order to limit the enzyme leakage observed after simple adsorption, for a proteomic workflow a very simple and fast procedure would be high desirable for digestion of single protein or complex protein mixture before MS analysis.

In conclusion, in our opinion, in the case of proteomic analysis the ease of the physical immobilization procedure compensates for the leaching of the enzyme which, obviously, decreases the number of rounds of reusability.

A critical issue in assessing the efficiency of such proteolytical meso-reactors, is to prove that an effective adsorption occurs inside the nanopores, with no leaching and/or non-specific surface sorption on outer surface of MPSs. Regarding this issue, it has been reported that when the mesopore diameter is sufficiently large to accommodate biomolecules, proteins penetrate into the mesoporous networks, as well as being adsorbed onto the external surface [25,29]. In fact when hexagonal mesoporous silicate with a pore size smaller than trypsin dimension was used as mesoreactor for protein digestion, the efficiency of proteolysis was drastically lowered in comparison to digestion performed in MPS with pore size larger than trypsin dimension and was only a little higher compared to that in bulk solution. To explain these data the authors hypothesized that *in mesopore* digestion did not occur because the pores of hexagonal mesoporous silicate were too small for entrapping trypsin in the internal surface [29].

Shui and colleagues observed a poor *in mesopore* digestion for conalbumin (pI 6) when protein loading in FDU-12 was performed at pH of 8.5 [27]. In fact at this operative pH both protein and silicate surface are both negatively charged, therefore electrostatic repulsion prevented substrate entrance in the mesochannels and proteolysis did not occur. An important concern regarding this point is the great contribution of surface derivatization to modulate electrostatic interactions between biomolecules and MPS. Depending on the enzyme/substrate or in general protein chemical-physical properties (idrophobicity, pI) by the means of appropriate functional groups grafted on the surface of MPS, it is possible to improve the driving force (electrostatic interactions) for protein diffusion and adsorption in the inner mesoporous networks thus providing a beneficial microenviroment for the hosted biomolecule [53,58,64]. Additionally, by studying the different surface properties of SBA-15 materials, Fan and colleagues ascribed to the “surface-character effect” the different performances of the host materials as proteolytical mesoreactors [25]. Interestingly, a poor digestion performance was observed for the SBA-15 material in which removal of the organic template was performed by calcination. On the contrary, the highest catalytic efficiency was observed for thiol functionalized SBA-15 material and intermediate activity was obtained for SBA-15 material in which organic templates were removed by ethanol extraction. A possible explanation given by the authors of this study resides in the abundant hydroxyl groups lying on the mesochannel walls after ethanol extraction. It could be possible that the interaction between substrates and silanol groups accelerate the unfolding process exposing more cleavage sites to the proteases [25].

In conclusion, although direct proof, for instance indisputable images of enzyme/substrate adsorbed inside the pore, is still missing, several pieces of circumstantial evidence strongly suggest that the improvement in proteolysis observed in the examples described so far can be ascribed to confinement in the mesopores with limited effect, if any at all, contributed by non-specific surface sorption on outside of mesoporous particles.

In the last few years the use of MPS for harvesting low molecular weight proteome from complex biological matrix such as plasma [52,53,54,104,105,106,107], serum [108], urine [53], saliva and induced sputum [109], has increased progressively. The simple and fast extraction protocols, the high sensitivity, reproducibility and the high capacity of the MPS are surely the keys to the success of this upcoming separation technology. Therefore we argue that in the nearly future the fruitful combination of proteins/peptide enrichment with the *in mesopore* protein digestion might serve as novel useful strategies for a deeper and high throughput proteomic analysis.

The design of MPS material to enhance protein digestion efficiency, although at its beginnings, is quite promising and the array of possible applications in proteomics are quite broad:

(1) First of all, efficient proteolysis in real time may open new horizons to high-throughput protein analysis. This is an essential feature, especially in the field of clinical applications of proteomics, where a lot of specimen need to be daily and routinely analyzed.

(2) In all the mesoreactors reviewed here, (i) only a small amount (few mg) of mesoporous silica particles is needed for digestion (Table 2); (ii) proteolysis takes place in few minutes (Table 2); (iii) the mesoreactors do not necessitate the manual sample handling and the extra steps necessary for *in gel* or *in solution* digestion, which are time consuming and lead to the introduction of contaminants such as human keratins.

These observations should encourage the miniaturization and the automation of the full process, from digestion to protein identification, without intermediate steps, which should dramatically lower even further its costs.

## 5. Conclusions

Confinement from molecular crowding in biological cells can both stabilize and induce order-of-magnitude enhancements in catalysis for large molecules and proteins, compared to enzyme reactions in solution. MPS offers an excellent platform to mimic the confinement present in such biological systems. In fact, the presence of an ordered array of pores (whose diameter, geometry and chemical functionalities can be finely tuned), as well as the high mechanical stability, create the optimal environment to host enzyme and substrate in a cell-like fashion.

Since the era of mesoreactors for proteolysis is just at its infancy, there are many open questions about the mechanisms which possibly regulate catalysis of enzymes entrapped in such supports. New studies will be required to better address the main factors which could affect and regulate proteolytic enzyme efficiency and stability in mesoporous support. Additional experiments are needed to better understand which factors may enhance the stability and reusability of enzymes in mesopores.

In our opinion a new forthcoming strategy in proteomics is shaping in the era of nanobiotechnology. Based on the use of immobilized protease reactors, in a near future, MPS nanobiocatalysts as “packed beads”, could be integrated into multidimensional separation systems for automated proteomics. Due to recent progress in the field of mesostructured silica materials, new synthetic routes will offer the opportunity to create the ideal mesospace suitable for stable enzyme-substrate entrapment. The ability to address these issues will ultimately determine how deeply material science, in particular nanotechnology, can contribute to satisfy the urgent and challenging task of proteomics.
